# Diagnostic performance of magnetic resonance imaging and ultrasonography on the detection of cesarean scar pregnancy: A meta-analysis

**DOI:** 10.1097/MD.0000000000027532

**Published:** 2021-12-03

**Authors:** Xiaoyi Xiao, Rixing Ding, Lei Peng, Huaping Liu, Yun Zhu

**Affiliations:** a Department of Radiology, The Third Xiangya Hospital, Central South University, Changsha, Hunan, People's Republic of China; b Department of Ultrasound, The First Hospital of Hunan University of Chinese Medicine, Hunan University of Chinese Medicine, Changsha, Hunan, People's Republic of China.

**Keywords:** cesarean section, ectopic pregnancy, magnetic resonance imaging, meta-analysis, ultrasonography

## Abstract

**Background:**

: There is still a debate on which imaging method is the best to diagnose cesarean scar pregnancy (CSP). Accordingly, this study aimed to analyze the diagnostic performance of magnetic resonance imaging (MRI) and ultrasonography (US) on the detection of CSP based on current evidence in the literature.

**Methods:**

PubMed, Embase, Cochrane, Chinese Biomedical Documentation Service System, WanFang, and China National Knowledge Infrastructure databases were searched up to June 2020. The included studies were all comparisons of MRI and US in the diagnosis of CSP that adopted postoperative histological examination as the reference standard. The pooled sensitivity, specificity, positive likelihood ratio (PLR), negative likelihood ratio (NLR), and area under the summary receiver operating characteristic curve (AUC) were calculated for MRI and US.

**Results:**

Thirteen studies were included, with a total sample size of 948 patients. The pooled sensitivity, specificity, PLR, NLR, and AUC of MRI in diagnosing CSP were 0.93 (95% CI, 0.91-0.95), 0.83 (95% CI, 0.75-0.89), 5.46 (95% CI, 3.70-8.05), 0.08 (95% CI, 0.06-0.11), and 0.96 (95% CI, 0.93-0.97), respectively; for US they were 0.84 (95% CI, 0.79-0.88), 0.73 (95% CI, 0.62-0.81), 3.06 (95% CI, 2.22-4.21), 0.23 (95% CI, 0.18-0.28), and 0.86 (95% CI, 0.83-0.89), respectively.

**Conclusion:**

We found that both MRI and US effectively diagnosed CSP; however, MRI had a higher diagnostic performance in detecting CSP than US.

## 1 Introduction

Cesarean scar pregnancy (CSP), a rare type of ectopic pregnancy, refers to the implantation of the gestational sac at the scar site from a previous uterine incision. It is one of the long-term complications associated with cesarean section.^[1]^ Recently, the incidence of CSP has increased to approximately 1/1800 to 1/2226, and it constitutes 6.1% of ectopic pregnancies in patients with a history of at least 1 cesarean delivery.^[2,3]^ The worldwide incidence of CSP has been rising, which may be attributed to the high rate of cesarean section and the rapid development of ultrasound technology.^[4–6]^ Besides, it is rather remarkable that according to the World Health Organization (WHO),^[7]^ the cesarean-section rate in China has reached 46.2%, which is among the highest in Asian countries.

There are many treatment options for women with CSP, but medications and surgery are still the most effective. Besides, a unified consensus on the standardized treatment of CSP has not yet been formed.^[8]^ If CSP is misdiagnosed or missed by the physician in the early stage,^[9]^ the patient will suffer serious complications such as uterine rupture, massive hemorrhage, and even death in the later stage.^[10,11]^ Therefore, early diagnosis and effective treatment are key for reducing these risks in women with CSP.^[12]^

The common symptom for CSP patients is usually little vaginal bleeding, occasionally accompanied by mild abdominal pain but most women are asymptomatic. So, diagnosis is mainly based on imaging examinations, namely, ultrasonography (US) and magnetic resonance imaging (MRI).^[13–15]^ Currently, US is the first-line imaging method for early diagnosis of CSP due to its low cost, short examination time, and high reproducibility.^[16]^ Simultaneously, it is necessary to strictly follow the diagnostic criteria of CSP to avoid misdiagnosis or miss diagnosis.^[17]^

However, due to the advancement of MRI technology today, MRI can clearly show the implantation site of the CSP gestational sac, the depth of the muscular layer invasion, and the relationships between adjacent tissues and cesarean scar, which are of high clinical value for diagnosis and subsequent treatment options.^[18,19]^ According to published articles,^[5,20]^ the diagnostic efficiencies of the 2 imaging methods are very high, but, to the best of our knowledge, no large series has yet compared MRI and US for CSP.

Therefore, the study aims to analyze the diagnostic performance of MRI and US for CSP by pooling relevant studies and to provide a potential basis for clinical diagnosis and treatment.

## 2 Methods

### 2.1 Literature search

This meta-analysis was implemented according to the preferred reporting items for systematic reviews and meta-analyses statement.^[21]^ PubMed, Embase, Cochrane, Chinese Biomedical Documentation Service System, China National Knowledge Infrastructure, and WanFang databases were searched up to June 2020. The search terms included “cesarean scar pregnancy/CSP”, “magnetic resonance imaging/MRI”, and “ultrasound”; the logical conjunction word was “and”. Besides, references were searched to identify additional relevant articles. Two reviewers independently screened all the research projects that were searched.

### 2.2 Inclusion and exclusion criteria

The inclusion criteria were as follows: a comparison of the diagnostic performance of MRI and US for CSP; a prospective or retrospective study; the diagnosis of CSP was based on postoperative pathological examination as the reference standard. The exclusion criteria were as follows: sample size <20 cases; incomplete data; repeated reports; and nonhuman research.

### 2.3 Data extraction and quality assessment

The main information in the study was extracted by 2 reviewers according to a predesigned data extraction table, including basic research information (first author of the study, publication year, number of sample cases, country, study design, age, pregnancy duration, and cesarean-section interval; diagnostic indicators for MRI and US and their corresponding 4-grid table data: namely, true positive (TP), false positive (FP), false negative (FN), and true negative (TN).

The quality assessment diagnostic accuracy studies-2^[22]^ was used independently by 2 researchers to evaluate the quality of the included studies, that is, to evaluate the risk of bias and clinical applicability of all items. Each item was categorized as “yes”, “no”, and “unclear”. If a disagreement was difficult to resolve, it was left to a third investigator to decide.

### 2.4 Statistical analysis

Stata version 16.1 and Meta-Disc 1.4 were used to perform this meta-analysis. Diagnostic performance was evaluated using the pooled data, including sensitivity, specificity, diagnostic odds ratios (DOR), positive likelihood ratio (PLR), negative likelihood ratio (NLR), and 95% confidence intervals (95% CIs).

For the sensitivity and specificity, a Spearman rank correlation coefficient *P* > .05 indicated there was no threshold effect; If *P* > .05, a bivariate random-effects model (Dersimonian-Laird assumption) was used to calculate pooled estimates of sensitivity, specificity, and DORs.^[23]^ Z-test is used for the discrimination of the 2 DORs. Positive and negative likelihood ratios (LRs) were used to characterize the clinical utility of the test and to evaluate the post-test probability of the disease.^[24]^ Using the average prevalence of the disease as the pre-test probability combined with the evaluation method and LRs, a Fagan plot was drawn to show the post-test probability.

Forest plots were used to display sensitivity, and specificity along with *I*^*2*^ and *χ*^*2*^ levels was obtained. Cochran *Q* test and *I*^*2*^ index were used to assess the heterogeneity of the sensitivity and specificity.^[25]^*P* values <.05 indicated heterogeneity. The *I*^*2*^ index was used to explain the degree of heterogeneity: when *I*^*2*^ > 50%, the heterogeneity was considered high; otherwise, the heterogeneity was considered low. Sensitivity analysis was used to verify the robustness of the pooled results, and a meta-regression analysis was used to explore the source of heterogeneity.

We plotted the summary receiver operating characteristic curve and calculate the area under the curve (AUC), compared the values for MRI and US in the diagnosis of CSP, and analyzed the diagnostic efficiency of the 2. A 2-tailed *P* < .05 was considered significant. Finally, a Deek funnel plot was used to evaluate whether the included studies had publication bias; A *P* < .10 indicated publication bias.^[26]^

### 2.5 Compliance with ethical guidelines

This is a meta-analysis involving data that were extracted from previously published original studies. In addition, our study was approved by the Ethics Committee of the Third Xiangya Hospital of Central South University, Changsha, China.

## 3 Results

### 3.1 Study search

**Figure 1:**
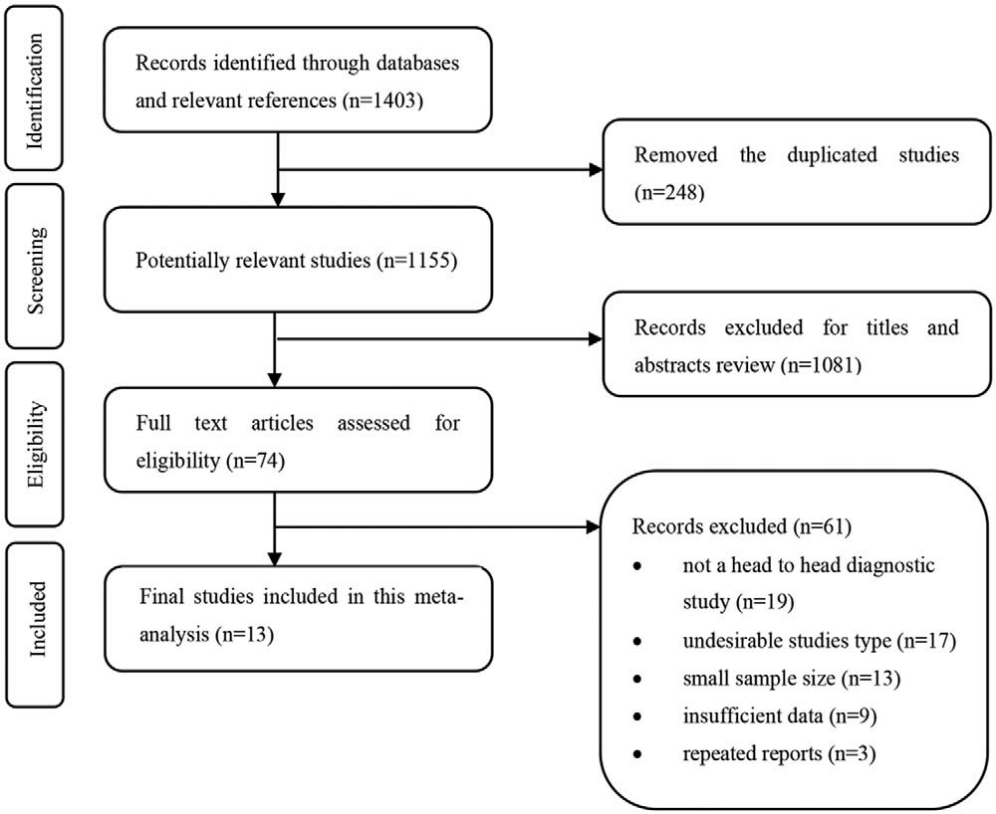
Selection process for studies included in the meta-analysis.

A preliminary search of 1403 studies from various databases was performed. After excluding 248 duplicate studies, 1155 studies remained. Then, 74 studies remained after screening the titles and abstracts. Finally, 13 studies^[27–39]^ that met the inclusion and exclusion criteria were included in the full-text screening. The screening process is shown in Figure 1.

### 3.2 Clinical characteristics of included studies

The 13 studies were retrospectively examined. The included patients were all from China, and the total sample size was 948 patients, with an average age of approximately 31.6 years. Most of the included studies were published within the last 3 years. The basic characteristics and diagnostic indicators of the included studies are shown in Table 1.

**Table 1:** Basic characteristics of included literature.

Study	Study design	No. of patients	Country	Mean age (yrs)	Pregnancy duration (mean ± SD, d)	CS interval (mean ± SD, yrs)
Liu, (2014)^[27]^	Prosp	81	China	32.3	30-84	NR
Chen, (2016)^[28]^	Prosp	28	China	30. 3	NR	1-5
Song, (2017)^[29]^	Prosp	97	China	30.5	63. 5 ± 10.2	3. 3 ± 1.3
Zeng, (2018)^[30]^	Prosp	56	China	34.5	38-106	1-4
Zhang, (2018)^[31]^	Prosp	68	China	NR	35-82	NR
Yang, (2018)^[32]^	Prosp	96	China	35.8	54.2 ± 10.6	2.2 ± 0.6
Gao, (2019)^[33]^	Prosp	90	China	29.5	61.5 ± 12.2	6.5 ± 1.2
Jin, (2019)^[34]^	Prosp	66	China	30.3	NR	NR
Li, (2019)^[35]^	Prosp	43	China	30.9	54. 6 ± 10.5	3.5 ± 1.0
Song, (2019)^[36]^	Prosp	163	China	33.5	40-75	4.9 ± 1.1
Zhan, (2019)^[37]^	Prosp	50	China	29.9	65.5 ± 18.5	3.3 ± 1.3
Li, (2020)^[38]^	Prosp	50	China	30.5	65.5 ± 10.9	3.3 ± 1.9
Liang, (2020)^[39]^	Prosp	60	China	29.7	47.4 ± 5.0	NR

The risk of bias and clinical applicability of the included studies are shown in Table 2. In terms of risk of bias in patient selection, there were 6 studies^[27,30,33,35–37]^ that did not report the inclusion criteria in detail. There was 1 study^[28]^ with a high risk of bias because it included a small number of cases, making it easy to overestimate the diagnostic performance. Moreover, there were 6 studies^[33–35,37–39]^ that did not describe the contents of the index test or its specific operations. As for the reference standard as well as the flow and timing, the included studies had relatively small risks of bias. There were 6 studies^[27,28,30,31,38,39]^ that did not elaborate on the details of patient selection. There was 1 study^[34]^ that failed to report most clinical characteristics. It should be noted, however, that index tests and reference standards for clinical applicability are generally of low concern.

**Table 2:** Results of QUADAS-2 assessment of the 13 included studies.

	Risk of bias				Applicability		
Study	Patient selection	Index test	Reference standard	Flow and timing	Patient selection	Index test	Reference standard
Liu, (2014)^[27]^	U	L	L	L	U	L	L
Chen, (2016)^[28]^	H	L	L	L	U	L	L
Song, (2016)^[29]^	L	L	L	L	L	L	L
Zeng, (2018)^[30]^	U	L	L	L	U	L	L
Zhang, (2018)^[31]^	U	L	L	L	U	L	L
Yang, (2018)^[32]^	L	L	L	L	L	L	L
Gao, (2018)^[33]^	U	U	L	L	L	L	U
Jin, (2019)^[34]^	L	U	L	L	H	L	L
Li, (2019)^[35]^	U	U	L	L	L	L	U
Song, (2019)^[36]^	U	L	L	L	L	L	L
Zhan, (2019)^[37]^	U	U	L	L	L	L	L
Li, (2020)^[38]^	L	U	L	L	U	L	L
Liang, (2020)^[39]^	L	U	L	L	U	L	L

### 3.3 Diagnostic performance of MRI and US in CSP

#### 3.3.1 Heterogeneity test

MRI: According to the Spearman rank correlation coefficient (–0.357, *P* = .237), there was no threshold effect. As shown in Table 3, the homogeneity tests for pooled sensitivity and specificity showed Q = 12.49 (*P* = .41), *I*^*2*^ = 3.89% and Q = 15.42 (*P* = 0.22), *I*^*2*^ = 22.18%, respectively, which showed the data had no obvious heterogeneity.

**Table 3:** Pooled sensitivity and specificity of MRI and US.

	Pooled sensitivity			Pooled specificity		
Diagnosis method	Q	*P*	*I* ^2^	Q	*P*	*I* ^2^
MRI	12.49	.41	3.89%	15.42	.22	22.18%
US	31.29	<.05	61.69%	31.7	<.05	62.15%

US: According to the Spearman rank correlation coefficient (0.401, *P* = .174), there was no threshold effect. As shown in Table 3, the homogeneity tests for pooled sensitivity and specificity showed Q = 31.29 (*P* < .05), *I*^*2*^ = 61.69% and Q = 31.70 (*P* < .05), *I*^*2*^ = 62.15%, respectively, which showed the data had obvious heterogeneity.

#### 3.3.2 Pooled analysis of diagnostic accuracy

**Figure 2:**
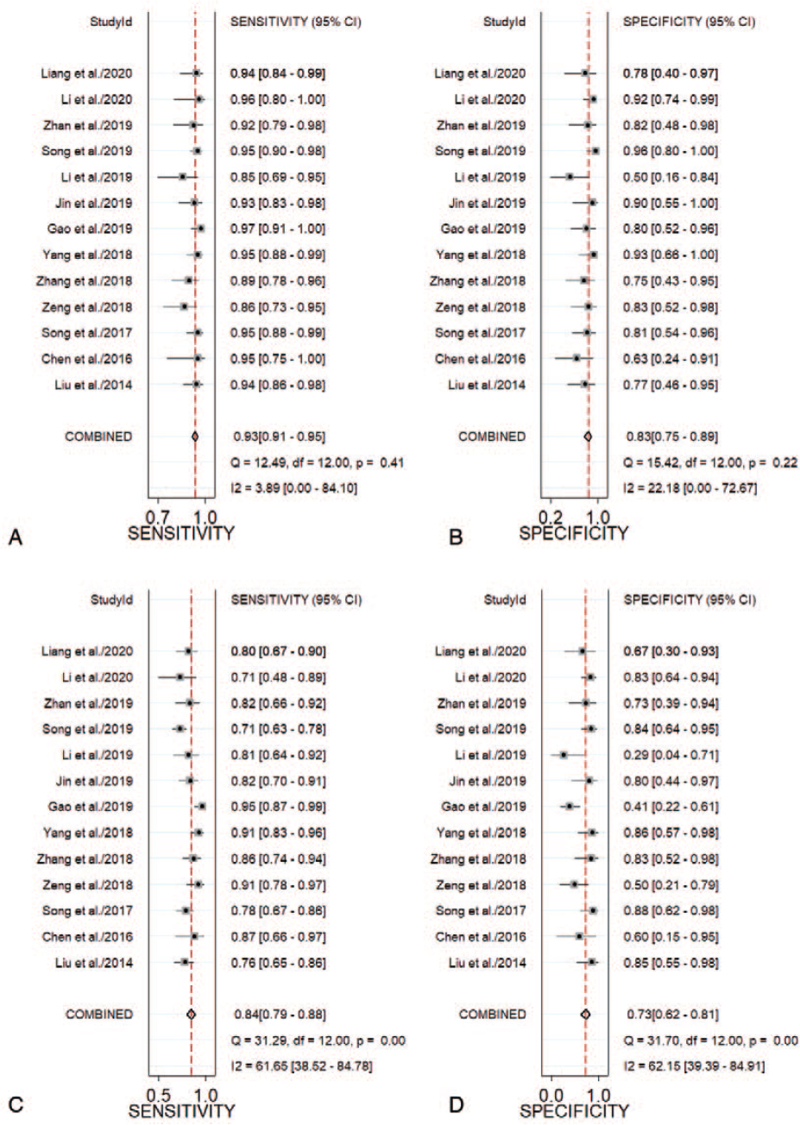
Forest plots of the sensitivities and specificities of MRI (A, B) vs US (C, D). CI = confidence intervals, MRI = magnetic resonance imaging, US = ultrasonography.

**Figure 3:**
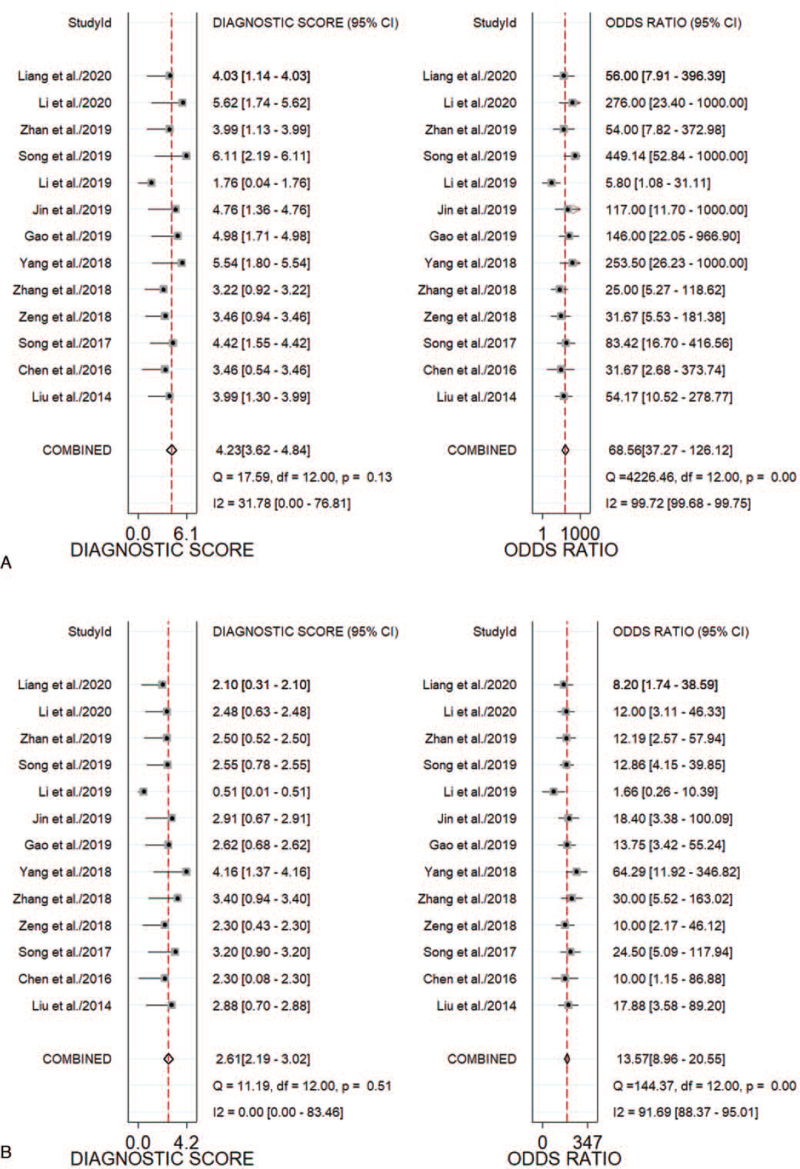
Forest plots of the DOR of MRI (A) vs US (B). CI = confidence intervals, DOR = diagnostic odds ratio, MRI = magnetic resonance imaging, US = ultrasonography.

As shown in Figure 2 (A, B), the pooled sensitivity and specificity of MRI in the diagnosis of CSP were 0.93 (95% CI, 0.91-0.95) and 0.83 (95% CI, 0.75-0.89), respectively. As shown in Figure S1 (A, B), Supplemental Digital Content the PLR and NLR results for MRI were 5.46 (95% CI, 3.70-8.05) and 0.08 (95% CI, 0.06-0.11), respectively. As shown in Figure 3 (A), the MRI DOR was 68.55 (95% CI, 37.27-126.1). As shown in Figure 2 (C, D), the pooled sensitivity and specificity of US in the diagnosis of CSP were 0.84 (95% CI, 0.79-0.88) and 0.73 (95% CI, 0.62-0.81), respectively. PLR and NLR were 3.06 (95% CI, 2.22-4.21) and 0.23 (95% CI, 0.18-0.28), respectively, in Figure S1 (C, D), Supplemental Digital Content. The US DOR was 13.57 (95% CI, 8.96-20.55) in Figure 3 (B).

**Figure 4:**
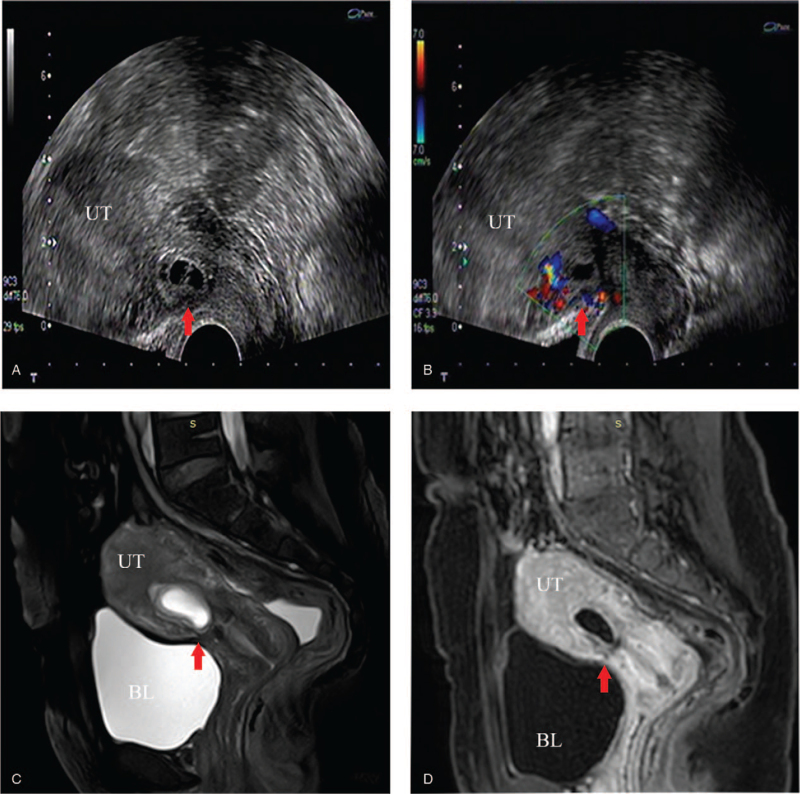
US and MRI images of CSP (from the author's institution). Transvaginal 2-dimensional grayscale (A) and color Doppler (B) ultrasound. Sagittal T2-weighted image (C) and sagittal T1-weighted image (D) of MRI. Gestational sac implantation site (red arrows). BL = bladder, CSP = cesarean scar pregnancy, MRI = magnetic resonance imaging, US = ultrasonography, UT = uterus.

**Figure 5:**
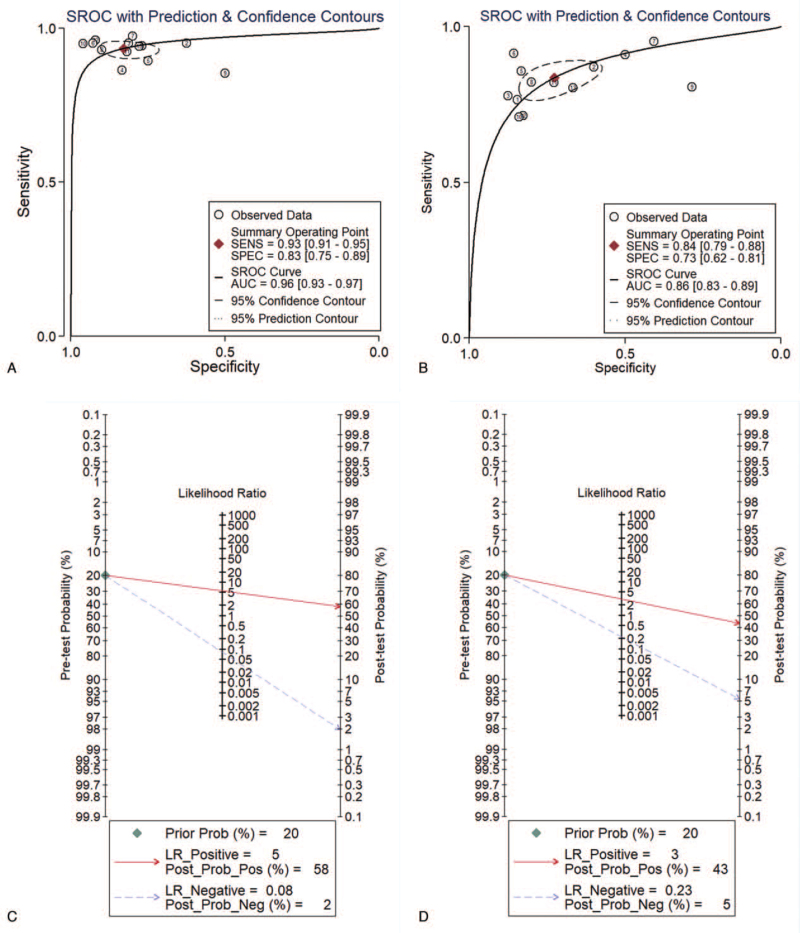
Summary of the ROC curves for MRI (A) vs US (B) with prediction and confidence contours. The diamond-shaped solid point is the summary operating point. The circle in the dashed line is the 95% confidence contour. Fagan nomogram for MRI (C) vs US (D). The pretest probability was fixed at 20%. AUC = area under the curve, MRI = magnetic resonance imaging, SROC = summary receiver operating characteristic, US = ultrasonography.

Besides, the Z-test results showed that the difference between the 2 diagnostic methods was significant (*P* < .05), which indicates the diagnostic performance of MRI for CSP was significantly better than that of US (Fig. 4). As shown in Figure 5 (A, B), the AUC for MRI was 0.96 (95% CI, 0.93-0.97); that for US was 0.86 (95% CI, 0.83-0.89). As shown in the Fagan nomogram in Figure 5 (C, D), the pre-test probability was 20%, the post-test probability of the positive predictive values of MRI and US increased to 58% and 43%, respectively, whereas the negative predictive values decreased to 2% and 5%, respectively.

### 3.4 Sensitivity and meta-regression analyses

For a sensitivity analysis, each of the 13 studies was excluded one-by-one. We found that when the study by Gao^[33]^ was excluded, the pooled sensitivity and specificity of the US group changed from 61.65% and 62.15% to 49.04% and 43.15%, respectively. Besides, when the study by Song^[36]^ was excluded, the pooled sensitivity changed to 44.79%. When the rest of the studies were excluded in turn, the results remained robust. Thus, the studies by Gao^[33]^ and Song^[36]^ may be the sources of heterogeneity. Meta-regression analysis of relevant factors such as the US group's sample size, description of the index test, and description of the reference standard showed that the source of heterogeneity could not be explained.

### 3.5 Publication bias

As shown in Figure S2 (A, B), Supplemental Digital Content Deek funnel plot asymmetry test showed significant publication bias for MRI (*P* = .001) while insignificant for US (*P* = .29) in the diagnosis of CSP (*P* < .10).

## 4 Discussion

Owing to the opening of the 2-child policy and the widespread application of US in China, reports of the incidence of CSP have increased.^[40]^ However, the precise incidence is still unknown, which may be attributed to potential underreporting and underdiagnosis.^[41]^ We need to know that many clinical cases have emphasized^[11,42]^ that most CSP patients are asymptomatic in the early stage when compared with non-ectopic pregnancy, which increased the difficulty of differential diagnoses. Also, as the pregnancy progresses, the mother's health and safety will be drastically affected by the development of the serious long-term complications of CSP.^[43,44]^ Therefore, early diagnosis and treatment are of particular significance. In clinical practice, it is important to identify whether a gestational sac in the lower uterus is CSP, as the appropriate treatment plan differs.^[45]^ At present, most early clinical diagnoses of CSP are based on imaging examinations,^[46]^ namely, MRI and US. However, the reference standard for diagnosing CSP is postoperative pathological examination.

This meta-analysis included 13 studies on the diagnosis of CSP by MRI and US, all of which were comparisons. Through this meta-analysis, the 2 imaging methods were compared to summarize and quantitatively analyze the relevant diagnostic performances and indicators of CSP. As shown in Table 1, most of the included studies were published in the past 3 years, and the included research populations were all Chinese. This indicates that the early diagnosis of CSP has received increased attention in recent years, especially in China, which reflects the high occurrence rate of CSP in China. As shown by the quality assessment diagnostic accuracy studies-2 evaluation items in Table 2, compared with other evaluation indicators, there are several studies judged to be “unclear” in terms of patient selection. This was because the provided information was not sufficient for us to judge it as “yes” or “no”. As shown in Figure 2, the pooled sensitivity and specificity of MRI/US were 0.93/0.83 and 0.84/0.73, respectively. Additionally, the Z-test demonstrated that MRI had a higher DOR than US (*P* < .01). As shown in Figure 5 (A, B), the AUCs of MRI and US were 0.96 and 0.86, respectively, indicating the diagnostic efficiency of both is high, but the diagnostic efficiency of MRI is higher than that of US (*P* < .01). As shown in Figure 5 (C, D), the PLR and NLR of MRI were 5 and 0.08, respectively, whereas those of US were 3 and 0.23, respectively. This indicates that US is of limited value in the clinical diagnosis and exclusion of CSP and needs to be combined with other clinical data to ensure proper analysis; thus, MRI is a better method in the clinical diagnosis of CSP. We found no obvious heterogeneity in the sensitivity and specificity of MRI in the diagnosis of CSP (*I*^*2*^ < 50%), whereas the heterogeneity in the sensitivity and specificity of US in the diagnosis of CSP was relatively high (*I*^*2*^ > 50%). Sensitivity analysis results showed the studies by Gao^[33]^ and Song^[36]^ to be potential sources of heterogeneity. Moreover, meta-regression analyses of the sample size, description of the index test, and reference standard of the US group showed that the source of the heterogeneity could not be explained.

US is the first-line imaging modality for evaluation of a potential CSP due to its advantages such as the use of nonionizing sound waves, being more readily available, being less expensive, and its real-time nature (Fig. 4A and B).^[47,48]^ With the advancement of imaging technology, the increase in the incidence of CSP, and the continuous emergence of complications, it has been found that the application of MRI in the diagnosis of such patients can significantly reduce the missed-diagnosis rate. However, MRI inspection takes longer, costs more, and has many contraindications compared with US, limiting its application. Thus, it is not suitable for large-scale clinical screening. According to the results of the present meta-analysis, the sensitivity and specificity of MRI diagnosis of CSP are better than those of US. The reasons for this difference may be as follows^[5,17,49]^: US is easily affected by the operator's clinical experience and awareness of CSP; soft-tissue contrast for ultrasound which is not that obvious when compared with MRI, thus making it difficult to determine whether the gestational sac is implanted at the site of a previous cesarean section scar. Moreover, it is also difficult to accurately determine the depth of implantation of the gestational sac into the muscle layer (Fig. 4A and B); MRI is a diagnostic method that can perform multi-planar and multi-sequence imaging with high spatial resolution, high soft-tissue resolution, and high blood flow sensitivity; and MRI can clearly show the relationships among the uterine cavity, cesarean section scar, and pregnancy sac through multidimensional images, further clarify the invasion of the gestational sac into the muscle layer and accurately measure the thickness of the isthmus (Fig. 4C and D).

This study did have some limitations. First, the number of CSP cases has increased, but it remains difficult to perform a prospective, large-sample study. Second, we only searched Chinese and English databases, and bias may have been introduced due to incomplete search data. Despite these limitations, our analysis can, to some extent, raise the awareness of clinicians to identify CSPs at an early stage, thereby reducing the pain and financial burden of misdiagnosis and omission on patients and providing potential support for the health of the general public.

## 5 Conclusions

In conclusion, our meta-analysis suggests that both MRI and US are effective for diagnosing CSP; however, MRI had a higher diagnostic performance in detecting CSP than US. More prospective, multicentre, large-sample, randomized controlled studies on MRI and US for CSP diagnosis are warranted.

## Author contributions

**Conceptualization:** Xiaoyi Xiao.

**Data curation:** Rixing Ding, Yun Zhu.

**Formal analysis:** Xiaoyi Xiao, Rixing Ding, Yun Zhu.

**Formal analysis:** Xiaoyi Xiao.

**Investigation:** Lei Peng.

**Methodology:** Xiaoyi Xiao, Lei Peng, Yun Zhu.

**Methodology:** Xiaoyi Xiao.

**Project administration:** Yun Zhu.

**Software:** Xiaoyi Xiao, Rixing Ding, Lei Peng, Huaping Liu.

**Software:** Xiaoyi Xiao.

**Validation:** Huaping Liu.

**Writing – original draft:** Xiaoyi Xiao.

**Writing – review & editing:** Rixing Ding, Yun Zhu.

## Supplementary Material

Supplemental Digital Content

## Supplementary Material

Supplemental Digital Content
