# Supplementary figures and images for: Diagnostic performance of magnetic resonance imaging and ultrasonography on the detection of cesarean scar pregnancy: A meta-analysis
Source: Medicine (Baltimore). 2021 Dec 3;100(48):e27532. doi: 10.1097/MD.0000000000027532 (PMC9191567; doi:10.1097/MD.0000000000027532)

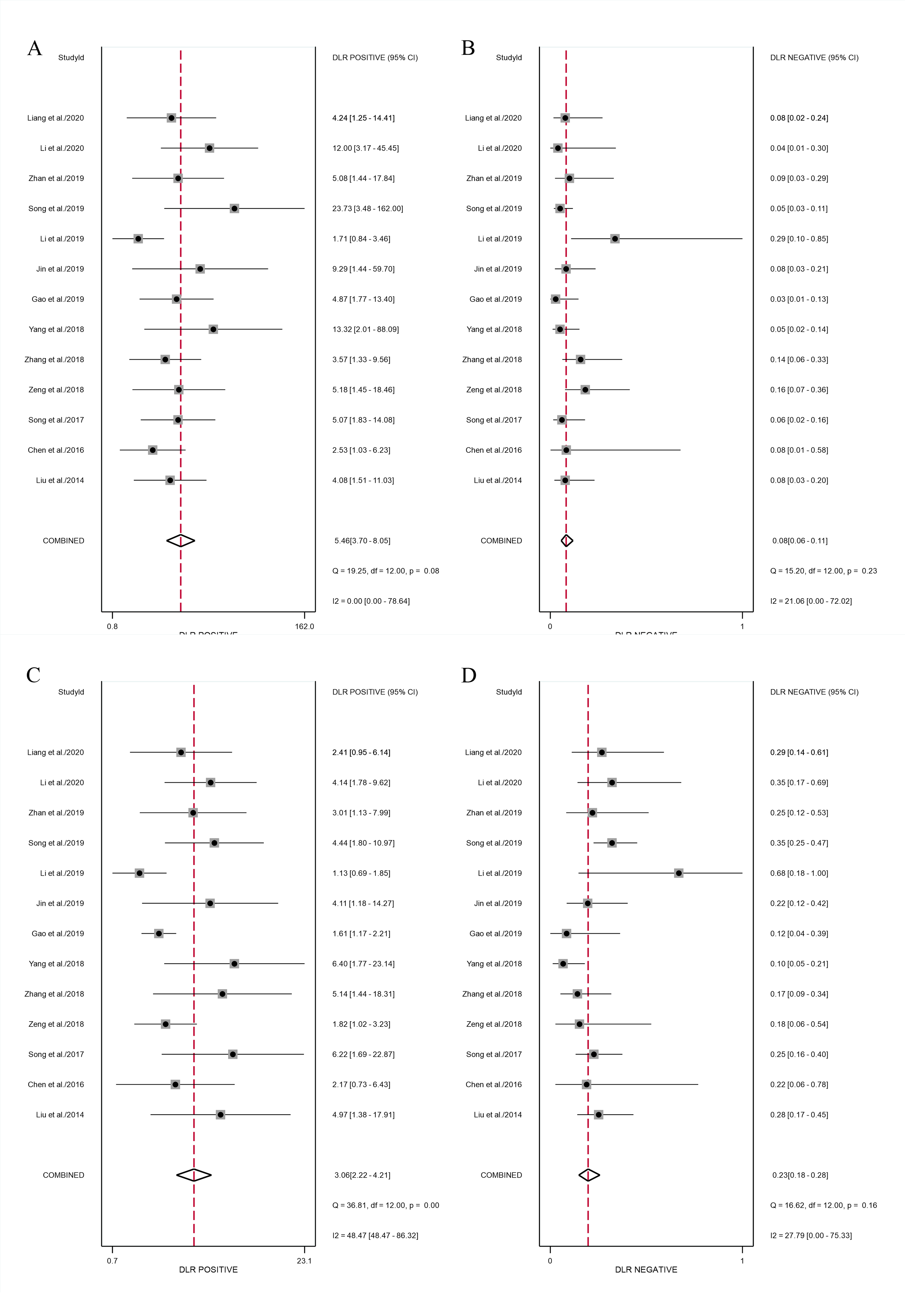


**Supplemental Digital Content (Figure. S1)** Forest plots of the PLR and NLR of MRI (A, B) vs. US (C, D)

Supplement: Supplemental Digital Content [file medi-100-e27532-s001.doc]
