# Supplementary material for: Diagnostic performance of magnetic resonance imaging and ultrasonography on the detection of cesarean scar pregnancy: A meta-analysis
Source: Medicine (Baltimore). 2021 Dec 3;100(48):e27532. doi: 10.1097/MD.0000000000027532 (PMC9191567; doi:10.1097/MD.0000000000027532)
Supplement: Supplemental Digital Content [file medi-100-e27532-s002.doc]

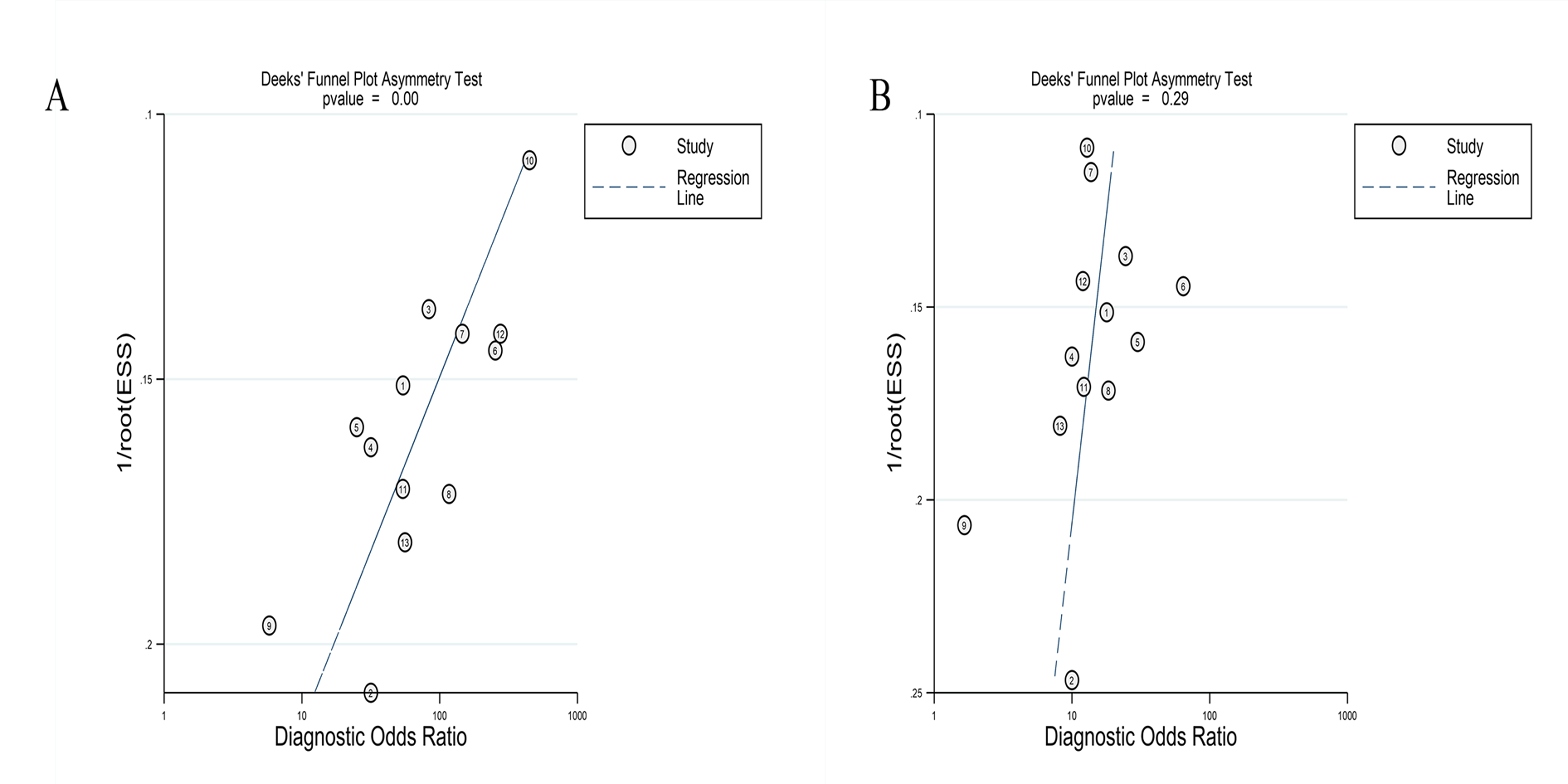


**Supplemental Digital Content (Figure. S2)** Deek’s funnel plot of publication bias on the pooled DOR of MRI (A) vs. US (B)
